# Bioactivity and osteointegration of hydroxyapatite-coated stainless steel and titanium wires used for intramedullary osteosynthesis

**DOI:** 10.1007/s11751-017-0282-x

**Published:** 2017-04-06

**Authors:** Arnold V. Popkov, Elena N. Gorbach, Natalia A. Kononovich, Dmitry A. Popkov, Sergey I. Tverdokhlebov, Evgeniy V. Shesterikov

**Affiliations:** 1 Laboratory for Limb Lengthening and Deformity Correction, Russian Ilizarov Scientific Center for Restorative Traumatology and Orthopaedics, 6 M. Ulianova Street, Kurgan, Russia; 2 Laboratory of Morphology, Russian Ilizarov Scientific Center for Restorative Traumatology and Orthopedics, 6 M. Ulianova Street, Kurgan, Russia 640014; 3 Head of Research Laboratory for Limb Lengthening and Deformity Correction, Russian Ilizarov Scientific Center for Restorative Traumatology and Orthopaedics, 6 M. Ulianova Street, Kurgan, Russia; 4 National Research Tomsk Polytechnic University, 30 Lenin Avenue, Tomsk, Russia

**Keywords:** Intramedullary osteosynthesis, Wire, Hydroxyapatite coating, Osteointegration, Bone formation, Osteosynthesis

## Abstract

A lot of research was conducted on the use of various biomaterials in orthopedic surgery. Our study investigated the effects of nanostructured calcium–phosphate coating on metallic implants introduced into the bone marrow canal. Stainless steel or titanium 2-mm wires (groups 1 and 2, respectively), and hydroxyapatite-coated stainless steel or titanium wires of the same diameter (groups 3 and 4, respectively) were introduced into the tibial bone marrow canal of 20 dogs (each group = 5 dogs). Hydroxyapatite coating was deposited on the wires with the method of microarc oxidation. Light microscopy to study histological diaphyseal transverse sections, scanning electron microscopy to study the bone marrow area around the implant and an X-ray electron probe analyzer to study the content of calcium and phosphorus were used to investigate bioactivity and osteointegration after a four weeks period. Osteointegration was also assessed by measuring wires’ pull-off strength with a sensor dynamometer. Bone formation was observed round the wires in the bone marrow canal in all the groups. Its intensity depended upon the features of wire surfaces and implant materials. Maximum percentage volume of trabecular bone was present in the bone marrow canals of group 4 dogs that corresponded to a mean of 27.1 ± 0.14%, while it was only 6.7% in group 1. The coating in groups 3 and 4 provided better bioactivity and osteointegration. Hydroxyapatite-coated titanium wires showed the highest degree of bone formation around them and greater pull-off strength. Nanostructured hydroxyapatite coating of metallic wires induces an expressed bone formation and provides osteointegration. Hydroxyapatite-coated wires could be used along with external fixation for bone repair enhancement in diaphyseal fractures, management of osteogenesis imperfecta and correction of bone deformities in phosphate diabetes.

## Introduction

Biomaterials are a variety of metallic components, polymers, ceramics or composite materials that are used and/or adapted for a medical application. A biomaterial may also be an autograft, allograft or xenograft used as a transplant material. A lot of research has been conducted to study the possible use of biomaterials in orthopedics that would provide bone fixation reinforcement, bone substitution or be able to induce new bone tissue formation and osteointegration [1, 2].

In order to improve bioactivity and osteointegration of metallic implants, their surface is coated with hydroxyapatite (HA) with the purpose of providing bonding with the surrounding bone tissue. It has been known that HA-coated hip implants used in hip arthroplasty showed bioactivity and osteointegration of their coating with the living bone tissue [3]. It was shown that primary implant stability is favored by HA coating which results in an improved contact between bone and implant. The studies demonstrated a sufficient osteointegration of HA-coated hip implants [4].

HA is thought to be an osteoconductive material that shows inductive ability when implanted extraskeletally [2]. It was also reported previously that even a non-soluble metal that contains no calcium or phosphorus can be an osteoinductive material when treated to form an appropriate macrostructure and microstructure [5].

Nailing and plating of a fractured bone are the most common methods in orthopedic trauma surgery. Recently, flexible intramedullary nailing with thin wires, HA coated or uncoated, has been advocated for management of diaphyseal fractures, lengthening and deformity correction [6–9]. It was observed that the use of such wires stimulated bone reparation and reduced the total period of treatment in clinical settings. Therefore, experimental studies were on the agenda to unveil these phenomena and obtain their explanations with fundamental research.

Our experiment was aimed at revealing the processes that undergo by introduction of HA-coated stainless steel or titanium wires into the bone marrow canal, and namely their bioactivity and osteointegration as compared with regular stainless steel or titanium wires.

## Materials and methods

The experiment was conducted on 20 dogs in the age between one and 3 years. Their mean body mass was 20 ± 2.9 kg.

Four types of wires were used for introduction into the bone marrow canal in dogs divided into four groups, each of five dogs. Groups 1 and 2 were dogs with regular stainless steel or titanium (Ti6Al4 V) wires, respectively (control groups). HA-coated stainless steel wires or HA-coated titanium (Ti6Al4 V) wires were introduced into the tibiae of study groups 3 and 4, respectively. HA coating on the wires was produced at the research laboratory of Tomsk Polytechnic University (Tomsk, Russia). The method of anode microarc oxidation (MAO) in an electrolyte that contained calcium (Ca) and phosphate (P) compounds was used to coat the surface of titanium wires (group 4). Stainless steel wires were first coated by a primary layer of titanium followed by microarc oxidation for HA coating (group 3). MAO processing results in mixing the oxidized substrate with the calcium and phosphate ions supplied from the electrolyte to form a Ca–P composite.

Microscopic study of wire surfaces was performed using a scanning electron microscope JSM-840 (JEOL, Japan). Calcium and phosphate concentrations in the coating were assessed using an X-ray electron probe analyzer INKA Energy 200 (Oxford Instruments Analytical, UK).

**Fig. 1:**
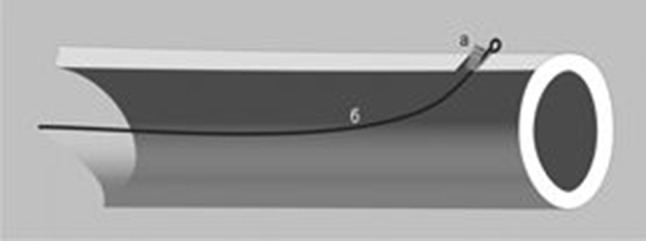
Fixation of an intramedullary wire with a formed end loop

The operations were performed by one surgical team. Under general anesthesia, one 2-mm wire with a slightly bent tip was introduced into the tibia from the medial side at the level of the tibial tuberosity from a tilted 3-mm hole preliminary drilled through the cortical layer into the bone canal and then was pushed down to the distal metaphysis. The opposite wire tip at the entrance point was cut, bent like a loop, placed under the segment fascia, and the soft tissues were sutured over it. Introduction of the wire did not required additional reaming (Fig. 1). The wires remained in the bone marrow canal during the entire experiment and were removed after four weeks.

Upon completion of euthanasia, 1-cm-high diaphyseal fragments were sawn out. They were fixed, decalcified and dehydrated according to a standard method and then embedded into celloidin. Transverse histotopographic sections, 20–24 µm thick, were obtained with a sledge microtome (Reichard, Germany), stained with hematoxylin and eosin and according to Van Gieson. Histological bone tissue preparations were studied with a large microscope (Carl Zeiss Opton, Germany). A software complex DiaMorph (DiaMorph, Russia) was used for morphometric study of the bone tissue content in the bone marrow canal and the thickness of the envelope formed around the intramedullary wire. Volume of bone tissue (%) in the digital images was defined with an analyzing software program VideoTest-Morfologia (St. Petersburg, Russia).

Concentrations of Ca and P in the transverse microsections of the diaphysis, embedded in araldite, were assessed using an X-ray electron probe analyzer INKA Energy 200 (Oxford Instruments Analytical, UK) that is adjusted to a scanning electron microscope JSM-840 under an accelerating voltage of 20 kV and operation state of 15 mm. The results were element maps and findings of their quantities in weight percentage.

Osteointegration was also assessed quantitatively by measuring the pull-off force with which the intramedullary wire was removed out of the medullary canal. The measurements were performed with a tensile sensor dynamometer DEPZ-1D-1U-1 (MeDeTal, Russia) that provides a measurement accuracy of ±0.01 N.

Statistical processing of the quantitative findings obtained was performed using a paired double-sample *t* test (*p* < 0.05) and Wilcoxon test for independent samples (*p* < 0.05).

The study was approved by the ethics board of the institution. Interventions, animal care and euthanasia conformed to the requirements of the *European Convention for the Protection of Vertebrate Animals used for Experimental and other Scientific Purposes* (Strasbourg, 18.03.1986); Principles of Laboratory Animal Care (NIH publication No. 85-23, revised 1985), as well as the national laws.

## Results

Clinical examination of the animals during the experiment did not reveal any changes in their general state, food or water consumption. No neurologic or infection complications were observed. Weight bearing was complete. Radiographic studies did not show any wire displacement in all the dogs.

### Microscopic study of wire surfaces

**Fig. 2:**
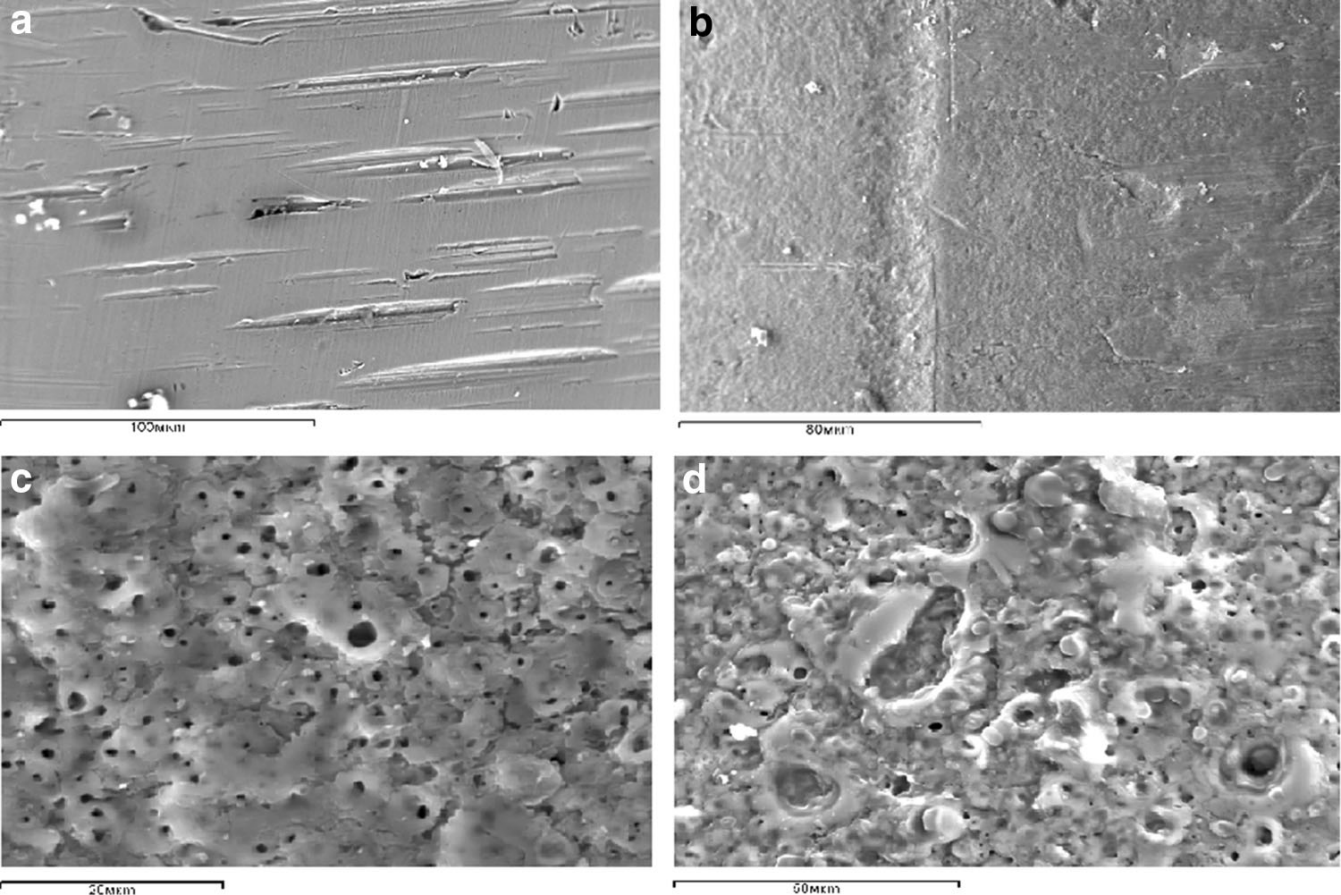
Surface microrelief: **a** SSW; **b** TW; **c** HA-coated SSW; **d** HA-coated TW. SEM, mag. **a** ×300; **b** ×600; **c** ×1600; **d** ×900

The surface of *a stainless steel wire* (SSW) featured transverse scratches, 7 × 2 µm long, 2 × 5 µm wide and 4 × 7 µm deep. The distance between the notches was from seven to 35 µm. The surface between the scratches was smooth (Fig. 2a).

The surface of *a titanium wire* (TW) featured a less expressed relief but a greater roughness that was uniform along its surface (Fig. 2b). The diameter of concaves and protuberances was from 0.4 to 1 µm.

The surface of *HA*-*coated wires* had round pours that were positioned on the entire surface of the coating and HA “waves.” The shape and location of such “waves” differed between the HA-coated SSW and HA-coated TW (Fig. 2c, d). Concentric spindles were formed round the pours on the surface of SSWs that united into a conglomeration, while the HA spindles on the TW surface were not linked to the pours but formed a trabecular-like picture where the crests of more protruded parts were interchanging with the pours and less protruded parts of HA coating. Globular structures that had a diameter from one to 4 µm were diffusely positioned on the coating of TWs. The pours of HA-coated wires also differed. HA-coated SSWs had pours that were from one to 4 µm in diameter with the distance of two to 7 µm between them. The pour openings on the TW coating were more variable with the diameter from one to 25 µm. The distance between them was not uniform and measured from two to 25 µm.

The content (weight percentage) of Ca, P and Ca/P ratio in the coating differed inconsiderably between the HA-coated wire types (Table 1). Concentrations of Ca and P were by 2.15 and by 5.7 lower in the coating of the TWs, respectively (*p* < 0.05). Ca/P ratio did not differ significantly (*p* ≥ 0.05).

**Table 1:** Content of osteotropic chemical elements in HA coating (wt%)

Element	HA-coated SSW	HA-coated TW
Ca	6.91	4.76
P	16.1	10.4
Ca/P	0.43	0.46

**Fig. 3:**
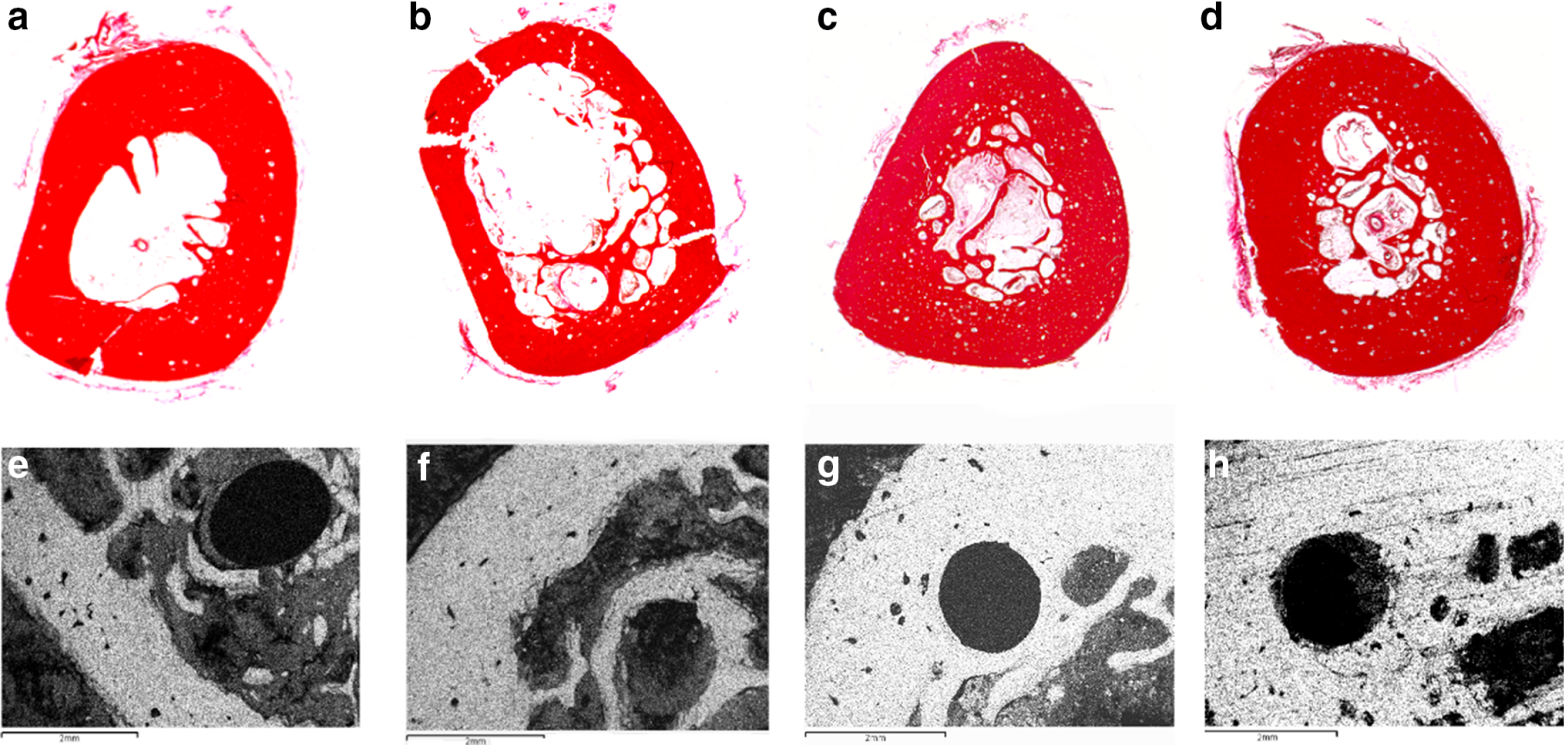
Osteointegration 1 month after intramedullary wires introduction: **a**, **e** group 1; **b**, **f** group 2; **c**, **g** group 3; **d**, **h** group 4. *Upper row* histotopographic sections. Hematoxylin and eosin staining. Mag. ×1.5. *Lower row* maps of X-ray electron probe microanalyzer by characteristic calcium radiation. Mag. ×20

We revealed significant differences in the diaphyseal bone structure between the groups after 4 weeks (Fig. 3). Widened Haversian canals were observed in the cortex of all dogs, but they were more expressed in groups 3 and 4. Trabecular bone in the bone marrow canal enveloped the wires like a muff and was seen in all the cases. An additional endosteal bone formation was also noted but was more expressed in groups 3 and 4.

Osteogenic activity was lower in group 1. Its envelope was discontinuous and was located over a connective tissue capsule. Its thickness ranged from 0.25 to 0.3 mm. Endosteal osteogenesis was week. The connective tissue capsule was absent in group 2, and the envelope thickness was between 0.4 and 0.6 mm. The endosteal response was more expressed than in group 1.

**Fig. 4:**
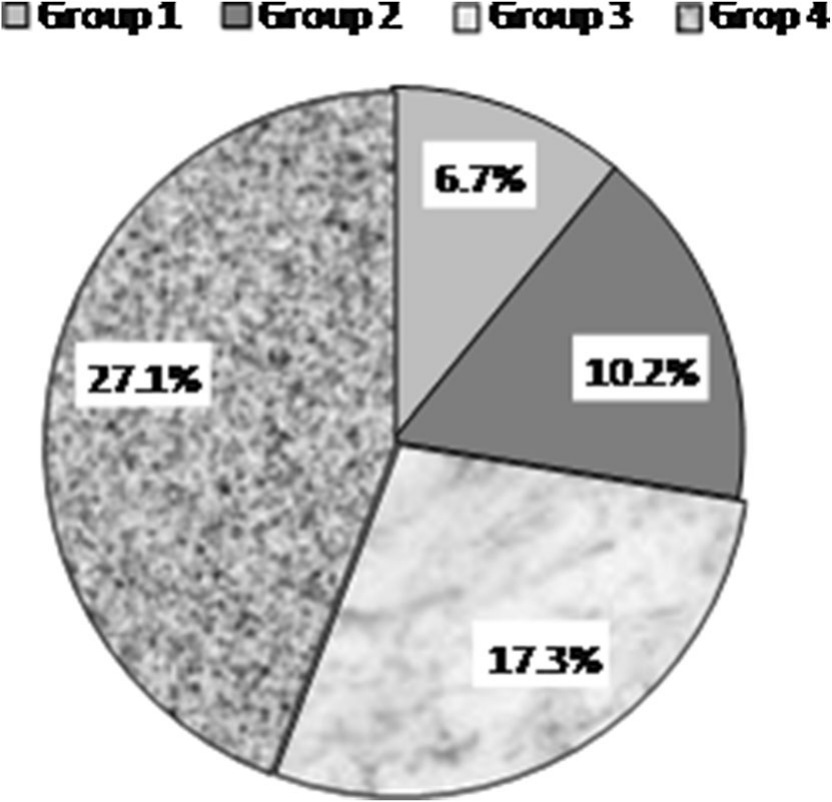
Percentage of bone tissue in the bone marrow of all groups canal of the tibia after 4 weeks of the experiment

The connective tissue capsule over the wires was absent in groups 3 and 4. HA-coated wires induced a greater endosteal osteogenesis as far as more volume of bone tissue was present in the bone marrow canal. The muff thickness was between 0.8 and 0.9 mm in group 3, while in group 4 it measured between 1.2 and 1.5 mm. Maximum percentage volume of trabecular bone was present in the bone marrow canals of group 4 dogs that corresponded to a mean of 27.1 ± 0.14% that was 16.9% higher than in group 2 (*p* < 0.05) (Fig. 4). It measured 17.3 ± 0.063% from the total volume of the bone marrow canal in group 3 dogs that was by 9.8% lower than in group 4 (*p* < 0.05) and by 10.6% lower than in control group 1 (*p* < 0.05).

The normal mean weight percentage of Ca in the canine cortex is 22%. The findings in Table 2 show that the outflow of Ca from the compact layer of groups 1 and 2 did not differ considerably as compared with groups 3 and 4 (2.5 and 2.28%, respectively). There was no considerable difference in the values between groups 3 and 4.

**Table 2:** Content of osteotropic elements in the tibia four weeks after the experiment (wt%)

Elements	Group 1		Group 2		Group 3		Group 4	
	Cortical bone	Cancellous bone of wire muff	Cortical bone	Cancellous bone of wire muff	Cortical bone	Cancellous bone of wire muff	Cortical bone	Cancellous bone of wire muff
Ca	**19.72**	*3.87*	**19.95**	*5.7*	20.2*	6.16*	21.92*+	8.76*
P	**9.02**	*1.96*	**9.15**	*2.75*	9.3*	3.07*	9.47*+	4.01*
Ca/P	**2.19**	*1.97*	**2.18**	*2.06*	**2.17**	**2**	2.3*+	2.18*

* *p* < 0.05 as compared with the wire type without HA coating

+ *p* < 0.05 as compared with the other HA-coated wire group

Italic values *p* < 0.05 as compared between the groups without HA coating

Bold type *p* ≥ 0.05

The content of osteotropic elements in the bony muff that was formed around the wires was the least in group 1 (Table 2). Weight percentage of Ca and P in the bone muff around TWs was higher in group 4. However, the Ca/P ratio did not have any statistical difference between TW groups. When groups 1 and 3 were compared, the content of Ca and P in the bone muff was increased 1.6-fold and 1.4-fold, respectively, in group 3. The increase in Ca and P was 1.54-fold and 3.44-fold higher in group 4 as compared with group 2, respectively. Moreover, the Ca/P ratio was close to a canine mature bone value in group 4.

Mechanical tests for measurements of the wire pull-off strength provided the quantitative data on the degree of wire osteointegration (Table 3). As it is seen in the table, the least pull-off force was observed by removal of the uncoated SSW. The microporous HA-coated TW surface increased the bonding force as compared with the uncoated TW. The force to pull out the HA-coated TW was 40% greater as compared with SSW in group 1.

**Table 3:** Pull-off strength of intramedullary wires

Strength (hPa)	Wire type			
	SSW	TW	HA-coated SSW	HA-coated TW
	351.1 ± 18	368.6 ± 15	421.0 ± 17*	494.7 ± 28*

* *p* < 0.05 as compared with a corresponding wire type without coating

## Discussion

All the metals and their alloys that have been used as implants or temporary fixators can be assessed in regard to their impacts on the living tissues. There are biotolerant materials such as stainless steel and cobalt chrome alloys, or bioinert materials such as titanium oxides and aluminum. Bioactive metals that would accelerate osteogenesis have not been known yet.

The main role of current plating and nailing methods is the mechanical retain of bone fragments in a reduced position until bone unites [10]. The loading is shunted to the plate or nail when a person ambulates. It is considered to be one of the drawbacks of these technologies as far as the main complications that develop are osteosynthesis instability and osteoporosis. Therefore, the solutions that would be able to enhance stability and bone reparation process by using nailing or plating have been under investigation [6, 10].

Ca–P ceramics (CaPC) and bioglass have been studied for possible use in bone tissue engineering. They have been characterized by tight chemical links with the bone (bone bonding) as well as by induction of bone formation on the implant surface [11, 12]. However, the CaPC that features an undoubted biological activity is too brittle to be used as a massive material of a sufficient volume that could endure much loading. Therefore, it cannot be applied as an independent implant [1]. It was found possible to coat the surfaces of metallic implants with CaPC or HA with the objective to combine the mechanical strength of the metal with the Ca–P bioactivity [11, 13–15]. The MAO technique enables to produce a qualitative and sufficiently thick nanocomposite HA coating on implants as compared with the RF sputtering technique that also reduces HA particles to nanoscale but a HA layer is of a lesser thickness [13–15]. The HA coating produced with the MAO technique on titanium surfaces has attractive properties, such as high porosity, a controllable thickness and a considerable density, which favor its use in dental and bone surgery [14, 15]. Moreover, the MAO technique is easier and cheaper for fabrication.

We have hypothesized that intramedullary osteosynthesis could be realized with HA-coated wires available on our market and started to use them instead of regular nails in clinical practice for reinforcement of bone fragments stability in diaphyseal fracture repair, bone lengthening and deformity correction in combination with the Ilizarov external fixation [6–9]. We observed that bone formation and repair went on faster with their use [7]. Therefore, we designed the experiment to study the effect of wires for diaphyseal fracture repair and found that facture healing was 1.6 times faster in the series with the combination of the techniques [6, 9]. The current study, being the continuation of the previous experiments of the possible ways to fasten osteointegration of wires, reveals not only the phenomena that undergo by introduction of the HA-coated wires into the medullary canal versus uncoated ones but also the degree of osteointegration by measuring the pull-off strength [10].

Microscopic, histological and biomechanical tests conducted during this experiment were aimed at an objective quantitative estimation of the HA-coated wires bioactivity in regard to osteoinduction and osteoconduction that may result in osteointegration. They revealed that the SSWs induced the least osteogenic activity in the bone marrow canal. It was found that HA coating stimulates bone formation around both SSWs and TWs. The implants were enveloped into an osseous muff and provided integration with bone tissue.

As the biomechanical tests showed [16], such wires got tightly fixed in the bone tissue of the muff formed around them. Other studies also assessed the bonding strength of various biomaterials with the bone and found that treatment methods are essential for preparing bioactive titanium [5, 17, 18]. In our opinion, the pull-off of the wire integrated into the bone tissue block is also influenced by the bonding of the coating with the wire surface. It may be conditioned by the features of the wire’s surface. We have revealed that the roughness that was more expressed in case of TWs as compared with the SSWs added to the bonding with HA coating. Finally, it contributed to a special “relief” of the surface due to coating that enhanced osteointegration properties of the titanium implant and formed a stronger implant-to-bone tissue block.

It is well known that one of the main drawbacks of the standard intramedullary osteosynthesis is the risk of damaging the medullary canal content, mainly its vessels [19]. It results in a weaker ability of pluripotent bone marrow stromal cells to osteogenesis and osteoinduction. As a matter of fact, the introduction of intramedullary wires almost excludes the damage to the intraosseous artery or injury to the endosteum as far as the wires we use have a bunt end, are of a small diameter (1.8–2.0 mm) and occupy not more than 30% of the canine bone marrow canal. In humans, they take a far more less space as even the narrowest part of the canal is wider. We did not have any arterial damage in our clinical practice [7, 8].

Moreover, a prolonged formation of local granulation foci in the bone marrow cavity could also have a stimulation effect. Local granulation foci that are created by endocrinic and paracrinic ways provide the increase in osteoproductive cells in the fracture area, stimulate regenerative angiogenesis and thus promote activation of osteoreparation. In such conditions, fracture repair runs faster and is of primary type without cartilaginous or connective tissue by bone bonding [3, 20]. The Ca/P ratio in the HA coating was less than 1.0. We believe that it also played a definite role in the launch of osteoconduction process as it was similar to the one that is observed by sedimentation of bone morphogenetic proteins on the collagen matrix, or in other words, to the initial stage of new bone tissue mineralization.

Being low invasive, the intramedullary osteosynthesis with HA-coated wires fulfills two main functions. First, it is able to provide an additional stability of bone fragments due to a fast osteointegration at the expense of the bony envelope formed. And second, it creates osteoconductive and osteoinductive conditions for bone tissue reparative regeneration due to a HA-based biomaterial. The method of intramedullary reinforcement with bioactive HA-coated wires stimulates the activity of bone marrow and provokes endosteal bone formation.

The performance of a HA-coated implant depends on its coating properties (thickness, porosity, HA content, crystallinity) and implant roughness [21]. It appeared that the HA-coated surface relief architecture and pours of our experimental wires did not provoke formation of connective tissue. Instead, the bone marrow canal was filled in with spongy bone which was sufficiently dense round the implant and provided its osteointegration.

We believe that the effect of such wires could be successively used for management of patients with diaphyseal fractures, osteogenesis imperfecta, phosphate diabetes, as well as in cases when large amounts of lengthening are required [20]. Further research and clinical tests in other orthopedic situations (bone defects and lengthening) are under way.

## References

[1] Campana V, Milano G, Pagano E, Barba M, Cicione C, Salonna G, Lattanzi W, Logroscino G (2014). Bone substitutes in orthopaedic surgery: from basic science to clinical practice. J Mater Sci Mater Med.

[2] Nakamura T (2007). Biomaterial osteoinduction. J Orthop Sci.

[3] Geesink RGT (2002). Osteoconductive coatings for total joint arthroplasty. Clin Orthop Relat Res.

[4] Zweymüller KA (2012). Bony ongrowth on the surface of HA-coated femoral implants: an x-ray analysis. Z Orthop Unfall.

[5] Fujibayashi S, Neo M, Kim HM, Kokubo T, Nakamura T (2004). Osteoinduction of porous bioactive titanium metal. Biomaterials.

[6] Popkov AV, Kononovich NA, Gorbach EN, Tverdokhlebov SI, Irianov YM, Popkov DA (2014). Bone healing by using Ilizarov external fixation combined with flexible intramedullary nailing versus Ilizarov external fixation alone in the repair of tibial shaft fractures: experimental study. Sci World J.

[7] Popkov AV, Popkov DA, Kononovich NA, EN Gorbach, Irianov YM, Tverdokhlebov SI, Bol’basov Yen (2016). Osteointegration of the intramedullary implant in fracture of the diaphysis of a long bone. J Glob Pharma Technol.

[8] Popkov D, Popkov A, Haumont T, Journeau P, Lascombes P (2010). Flexible intramedullary nail use in limb lengthening. J Pediatr Orthop.

[9] Popkov A, Aranovich A, Popkov D (2015). Results of deformity correction in children with X-linked hereditary hypophosphatemic rickets by external fixation or combined technique. Int Orthop.

[10] Starr AJ (2008). Fracture repair: successful advances persistent problems and the psychological burden of trauma. J Bone Jt Surg Am.

[11] Kitsugi T, Nakamura T, Oka M, Senaha Y, Goto T, Shibuya T (1996). Bone-bonding behavior of plasma-sprayed coatings of BioglassR, AW-glass ceramic, and tricalcium phosphate on titanium alloy. J Biomed Mater Res.

[12] Anselme K, Noel B, Hardouin P (1999). Human osteoblast adhesion on titanium alloy, stainless steel, glass and plastic substrates with same surface topography. J Mater Sci Mater Med.

[13] Hu CJ, Lu JR (2015). Preparation of hydroxyapatite-containing coatings on pure titanium by linearly increasing the voltage in the pulsed bipolar microarc oxidation process. Int J Electrochem Sci.

[14] Lugovskoy A, Lugovskoy S (2014). Production of hydroxyapatite layers on the plasma electrolytically oxidized surface of titanium alloys. Mater Sci Eng C.

[15] Malchikhina AI, Shesterikov EV, Bolbasov EN, Ignatov VP, Tverdokhlebov SI (2016). Hybrid calcium phosphate coatings for implants. AIP conference proceedings 1760: 1 http://aip.scitation.org/doi/pdf/10.1063/1.4960266

[16] Dhert WJA, Jansen JA, An YH, Draughin RA (2000). The validity of a single push-out test. Mechanical testing of bone and the bone-implant interface.

[17] Nishiguchi S, Nakamura T, Kobayashi M, Kim HM, Miyaji F, Kokubo T (1999). The effect of heat treatment on bone-bonding ability of alkali-treated titanium. Biomaterials.

[18] Fujibayashi S, Nakamura T, Nishiguchi S, Tamura J, Uchida M, Kim HM, Kokubo T (2001). Bioactive titanium: effect of sodium removal on the bone-bonding ability of bioactive titanium prepared by alkali and heat treatment. J Biomed Mater Res.

[19] Pfister U (2010). Reamed intramedullary nailing. Orthopade.

[20] Popkov D (2015). Combined stimulating methods reconstructive surgery in pediatric orthopedics.

[21] Blackwood DJ, Seah KH (2010). Influence of anodization on the adhesion of calcium phosphate coatings on titanium substrates. J Biomed Mater Res A..

